# Genetics of antigen processing and presentation

**DOI:** 10.1007/s00251-018-1082-2

**Published:** 2018-09-13

**Authors:** Adrian Kelly, John Trowsdale

**Affiliations:** Department of Pathology, University of Cambridge, Cambridge, CB21QP UK

**Keywords:** MHC, HLA, Antigen processing, Antigen presentation, Genetics

## Abstract

Immune response to disease requires coordinated expression of an army of molecules. The highly polymorphic MHC class I and class II molecules are key to control of specificity of antigen presentation. Processing of the antigen, to peptides or other moieties, requires other sets of molecules. For classical class I, this includes TAP peptide transporters, proteasome components and Tapasin, genes which are encoded within the MHC. Similarly, HLA-DO and -DM, which influence presentation by HLA class II molecules, are encoded in the MHC region. Analysis of MHC mutants, including point mutations and large deletions, has been central to understanding the roles of these genes. Mouse genetics has also played a major role. Many other genes have been identified including those controlling expression of HLA class I and class II at the transcriptional level. Another genetic approach that has provided insight has been the analysis of microorganisms, including viruses and bacteria that escape immune recognition by blocking these antigen processing and presentation pathways. Here, we provide a brief history of the genetic approaches, both traditional and modern, that have been used in the quest to understand antigen processing and presentation.

## Some history

The early history of the genetics of antigen processing and presentation followed from the work on histocompatibility in mice, initially by Peter Gorer, who worked at the Lister Institute and Guy’s hospital in London. His discovery of the MHC in turn was inspired by three developments. First was the curious pastime of inbreeding mouse strains, a fashionable hobby which spread from China. It reached America in the early 1900s, and after a while, pioneering geneticists realised the advantage of inbred strains for research. Many of the mouse strains were started over 100 years ago. C57BL, one of the original strains, was designated as the mice were black and were number 57. BALB/c mice were white, on the other hand, and were designated by their originator, Halsey J. Bagg, as Bagg albino, or BALB/c for short. A fascinating early history of these developments is presented in a book on the *natural history of the MHC* (Klein 1986).

Inbred strains were pivotal in the next development, where researchers used them to study the genetics of tumour rejection. As early as 1903, it was discovered that tumours that grew well when transferred within the same strain were rejected in a different one. Then, in 1922, Little and Johnson showed that transplantation of normal tissue was subject to the same strain specificity as tumours. A third stimulus was the development of blood group research, largely attributed to Landsteiner.

JB Haldane suggested that tumour resistance factors may be akin to blood group antigens, but it was Gorer who performed experiments to test the idea that antigens were shared by both malignant and normal tissues. This led to the formulation of an immunological theory of transplantation, which was later systematised by Peter Medawar. George Snell was studying similar phenomena, and after collaborating with Gorer, he proposed calling the tumour-resistance factors *Histocompatibility* genes. His approach started to reveal some of the complexity of histocompatibility.

Early work leading to the discovery of the *human* HLA complex developed in the 1950s and was dependent on the study of antibodies against alloantigens on white blood cells by three laboratories: Jean Dausset in Paris, Rose Payne and Walter Bodmer in Stanford, and Jon van Rood in Leiden. It was realised that some patients, and women who had borne several children, tended to make such antibodies, which were independent of ABO blood groups and erythrocytes. At the time, human organ transplantation was becoming widespread, and it gradually became accepted that human leukocyte antigens were the equivalent of mouse H-2 antigens. The hope was that careful matching, as in ABO, could lead to organ transplants that were not rejected. It was soon realised that the HLA system was more complex than ABO and progress depended on exchange of cells and antisera. The *International Histocompatibility Workshops*, which have been held every few years since 1964, were critical in interpreting and integrating information obtained with a variety of techniques from different laboratories. Analysis of data from these workshops indicated that a single genetic region was pivotal, namely, in humans, HLA, the Major Histocompatibility Complex. An associated protein chain, β2microglobulin, was identified as a component of HLA antigens and was later mapped outside the complex to chromosome 15 (Goodfellow et al. 1975).

Attempts to develop in vitro assays to study graft rejection led to the mixed lymphocyte reaction (MLR), which also turned out to be controlled by the MHC region. However, the results did not correlate completely with the serologically defined determinants. Work in both human and mouse indicated that these determinants, as well as MLR responsiveness, were both part of the H2 and HLA complexes but were separated genetically. Meanwhile, a different set of experiments showed that levels of antibody response to short synthetic polypeptides were controlled by the MHC. For example, C57 mice responded well to the branched synthetic polypeptide (T, G)-A-L, but CBA animals were poor responders. These effects were traced to the H-2 region leading to the so-called immune response (Ir) genes (Benacerraf and McDevitt 1972).

These many years of work, all indicated that the MHC was a major hub controlling a number of immunological phenomena. Indeed, additional experiments showed that the MHC also controlled susceptibility to viruses. Work in Canberra showed that cytotoxic T cells simultaneously recognised viral antigens and MHC molecules, in a phenomenon that came to be known as MHC restriction (Zinkernagel and Doherty 1974).

The nature of the MHC became even more complex when it was proposed that T cell suppression was also controlled by the class II region (Green et al. 1983). There followed a decade of controversy over the existence of this phenomenon (Bloom et al. 1992). It was eventually accepted, and the responsible T cells were called *regulatory* to distinguish them from the confusing history of suppression (Sakaguchi et al. 2007). The genetics behind the controversy concerned the I-J gene which was supposed to map to the I region of the mouse MHC and control the function of suppressor T cells. The I-J antigens were proposed to be soluble molecules secreted by suppressor T cells. It was a shock to find that discrete I-J genes did not exist once the I region of the mouse had been cloned (Kronenberg et al. 1983).

The genetics of antigen processing and presentation was advanced dramatically in the late 1980s when two further major technical developments helped to get to grips with the complexity. One was the DNA cloning revolution and the second was the determination of the structure of MHC molecules from crystals. Initial cloning of H-2 and HLA antigen genes led quickly to assembly of maps of the MHC in humans and mice. These dramatically simplified the picture to just a handful of class I and class II loci, albeit with a profound level of polymorphism. The early maps of mouse MHC were painstakingly assembled from overlapping cosmids (Steinmetz and Hood 1983). Many different human haplotypes have been analysed by these techniques (Horton et al. 2008; Shiina et al. 2004), as well as using more modern, high-throughput approaches (Norman et al. 2016).

The nature of the polymorphism was mysterious, as class I and class II chains encompassed many amino acid changes seemingly scattered throughout the first two domains of class I and the first domains of both chains of class II. The breakthrough came with the crystal structure of the first MHC antigen, HLA-A2 by Pamela Bjorkman, who was a PhD student at the time in the Wiley/Strominger laboratories (Bjorkman et al. 1987a, Bjorkman et al. 1987b). The realisation that class I and class II molecules possessed a groove which bound peptides immediately swept away other models of antigen recognition, such as those invoking independent receptors for antigen and histocompatibility molecules on T cells.

The development of molecular immunology through the creation of mutants, DNA sequencing, protein structure and gene discovery then paved the way for uncovering the various components of the antigen processing and presentation pathways, as outlined below (Table 1).

**Table 1:** Some human antigen processing and presenting components. Alternative names and gene designations are given in parentheses

Gene	Comments	Chromosome
HLA-A	Classical class I	6
HLA-B	Classical class I	6
HLA-C	Classical class I	6
HLA-E	Non-classical class I	6
HLA-F	Non-classical class I	6
HLA-G	Non-classical class I	6
MICA	Class I –related	6
MICB	Class I –related	6
CD1a	Presents lipids	1
CD1b	Presents lipids	1
CD1c	Presents lipids	1
CD1d	Presents lipids	1
CD1e		
MR1	Presents metabolites to MAIT cells	1
HLA-DR	Classical class II	6
HLA-DQ (HLA-MB, DC)	Classical class II	6
HLA-DP (HLA-SB)	Classical class II	6
HLA-DO (HLA-DNA, DZA + HLA-DOB)	Non-classical class II	6
HLA-DM (RING6 + RING7))	Non-classical class II	6
Invariant chain (CD74, Ii)		5
β2microglobulin (B2M)	Component of mature class I	15
LMP2 (PSMB9, RING12)	Inducible proteasome subunit	6
LMP7 (PSMB8, RING10)	Inducible proteasome subunit	6
MECL1 (PSMB10)	Inducible proteasome subunit	16
PA28a (PSME1)	Assembly of immunoproteasome	14
PA28b (PSME2)	Assembly of immunoproteasome	14
TAP1 (RING4, PSF1)	Peptide transporter subunit	6
TAP2 (RING11, PSF2)	Peptide transporter subunit	6
ERp57 (PDIA3, GRP58)	Oxidoreductase in TAP complex	15
ERAP1	Amino peptidase	5
Tapasin (TAPBP)	Peptide loader	6
TAPBPR (TAPBPL)	Peptide editor	12
BIP (HSPA5)	ER chaperone	9
GILT (IFI30, IP30)	Thiol reductase	19
PDI (P4HB)	ER chaperone (redox-regulated)	19
Calreticulin (CALR)	ER chaperone	19
Calnexin (CANX)	ER chaperone	5
UGT1 (UGT1A1)	Glucuronosyltransferase	2
CIITA	Master class II transcription factor	16
NLRC5	Master class I transcription factor	16

## Mutant cell lines

Panels of HLA homozygous typing cell lines have been important tools in the analysis of human tissue types through HLA classes I and II. These lines were derived by Epstein Barr Virus transformation of lymphoid cells from consanguineous mating, usually first cousin. They have been used for over 40 years by the HLA community and are still in use today (Turner et al. 2018).

**Fig. 1:**
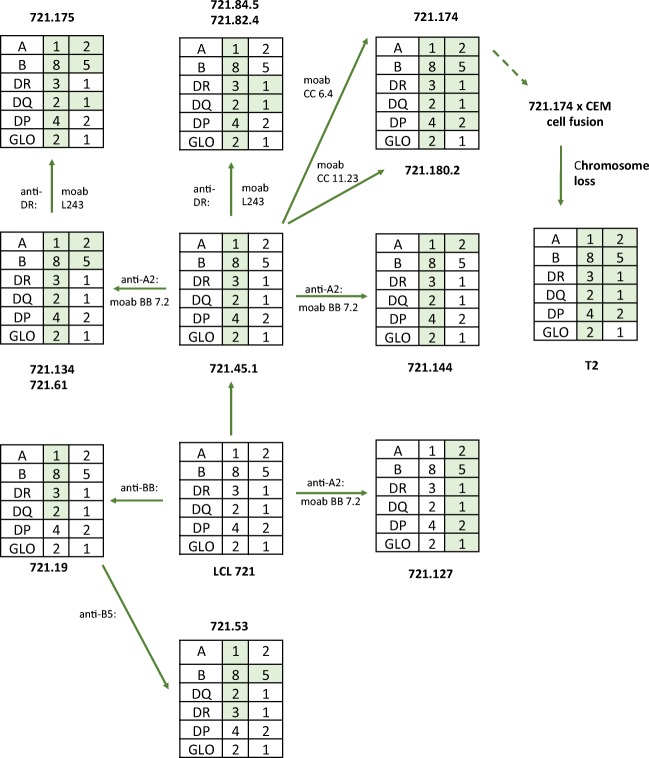
Composition of the mutant lines derived in the DeMars laboratory (DeMars et al. 1984; Kavathas et al. 1980) and the further derivative T2 (Salter et al. 1985). The shaded boxes show the proposed extents of the deletions. See text for more details of typing. These mutant cells have been used in numerous antigen processing and presentation studies. The Figure adapted from Demars et al. 1984

Analysis of the involvement of the HLA region in antigen processing and presentation was further enhanced by the generation of deletions and other mutations in lymphoblastoid cell lines (Fig. 1). One series of mutants was made by *γ* ray mutagenesis of the cell line B-LCL721 by the DeMars group (DeMars et al. 1984; Kavathas et al. 1980). Cells were subjected to 2 cycles of mutagenesis followed by immune-selection with monoclonal antibodies for MHC antigen loss. B-LCL721 contains the following two MHC haplotypes:

- HLA-A*01 HLA-B*08 HLA-DRB1*03 HLA-DPB1*04
- HLA-A*02 HLA-B*51 HLA-DRB1*01 HLA-DPB1*02

More details on the class I HLA types are available on the IPD-IMGT/HLA database.^1^ To our knowledge, not all class II genes have been typed in these cells. Mutant cell lines were isolated by subjecting these cells to irradiation. After 5 days growth, the survivors were selected with complement and a variety of monoclonal antibodies. Analysis of these mutants showed that they contained large homozygous or hemizygous deletions over the MHC region (Fig. 1). One such line LCL721.174, and its derivative 174xCEM.T2, has been used extensively to investigate TAP transporter function and MHC class I peptide loading. 174xCEM.T2, more commonly referred to as T2, was generated by fusing LCL721.174 with CEMR.3, a T-LCL, and selecting for loss of CEMR.3-derived copies of chromosome 6 (Salter et al. 1985). Both 174XCEM.T2 and LCL721.174 therefore carry the same chromosome 6 MHC deletion.

A similar procedure was used by Andreas Ziegler, using the lymphoma cell line BJAB.B95.8.6 (Spring et al. 1985). The wild-type cells were typed with the resolution available at that time as:

- HLA-A*1 HLA-B*35 HLA-C*4 HLA-DR*5 HLA-DQw3 HLA-DP*4
- HLA-A*2 HLA-B*13 HLA-C* HLA-DR* HLA-DQw1 HLA-DP*2

The Pious laboratory used a variation of this approach to isolate mutants in class II (8.1.6 and 9.28.6) by mutagenizing with EMS an HLA-DR3+ cell line, T5-1 (Mellins et al. 1988; Pious et al. 1985). Similarly, in mouse studies designed to investigate the nature of self and non-self-recognition, Klaus Karre generated a murine cell line RMA-S that lacked surface H-2 expression but was rejected in tumour transplant models (Karre et al. 1986). Analysis of this cell line was instrumental in providing early models for peptide loading by MHC class I molecules (Townsend et al. 1989). In addition, Roberto Accolla irradiated Raji cells to produce a mutant line RJ2.2.5 selecting against HLA-DR expression with an antibody D1–12(Accolla 1983), facilitating identification of CIITA.

These mutant cell lines and their derivatives have been instrumental in a long series of experimental approaches to uncover various components of antigen processing, and they continue to be important tools in investigating their mechanism of action.

## TAP transporters

A key insight into antigen processing was the finding that influenza A-specific cytotoxic T lymphocytes (CTL) recognise discrete epitopes of the nucleoprotein molecule. These data implied a mechanism for generation of viral protein fragments and their transport to the cell surface for presentation to CTL (Townsend et al. 1985). The peptide-binding groove in HLA molecules was a strong contender for the peptide carrier. There was a problem though in that MHC molecules were integrated into the cell membrane, so how did peptide fragments, which lacked signal sequences, access the endoplasmic reticulum (ER) to be loaded? How were the peptides transported to the cell surface on MHC molecules?

The solution came from MHC mapping and analysis of the mutants referred to above. Several groups simultaneously identified genes encoding transporter molecules mapping to the MHC. These were transporters of the so-called ABC (ATP-binding cassette) type, which were subsequently named TAP, for Transporters Associated with Antigen Processing (Deverson et al. 1990; Monaco et al. 1990; Spies et al. 1990; Trowsdale et al. 1990). These data suggested immediately that the transporters, encoded as a heterodimer of two proteins, TAP1 and TAP2, were responsible for pumping peptides into the ER (Kelly et al. 1992). To confirm this, the TAP1 sequence restored HLA class I expression when transfected into a cell line with a mutant TAP1 gene, LCL721.134 (Spies and DeMars 1991), and the TAP2 sequence restored function and co-precipitation of TAP1 and TAP2 when transfected into cells deficient in TAP2 (Kelly et al. 1992). However, this was not the case when using LCL721.174 cells, which maintained a large deletion over the class II region, covering both components of the heterodimer, TAP1 and TAP2(Spies and DeMars 1991). Studies of rats and chickens were particularly informative as the TAP genes are polymorphic in these species. It turned out that specific allelic versions of TAP supply peptides appropriate for class I molecules whose genes are linked in *cis* on the haplotype. Inappropriate pairing of TAP alleles with class I alleles they serve with non-binding peptides led to reduced expression of class I, which could be detected at the cell surface (Deverson et al. 1990; Kaufman 2015; Powis et al. 1996).

## Natural mutants in TAP

A number of individuals have been identified with immune-deficiencies due to mutations in TAP (de la Salle et al. 1994; Zimmer et al. 2005). These belong to the category of type I bare lymphocyte (BLS) syndrome, where HLA class I, but not class II, is affected. These patients generally have a severe reduction of class I at the cell surface, but in spite of this, most of these individuals reach adulthood. They are rare and are almost exclusively HLA homozygous due to first cousin parentage. They generally display chronic infections of the respiratory tract (purulent rhinitis, pansinusitis, otitis media) with bacteria (*H. influenzae*, *S. pneumoniae*, *S. aureus*, *K. pneumoniae*, *E. coli* and *P. aeruginosa*). About half of them exhibit granulomatous skin lesions. This may be due to suboptimal cytokine and cellular responses, leading to tissue damage and favouring subsequent bacterial infection and further recruitment of phagocytic cells. Indeed, antibiotics that impair neutrophils appear to be beneficial.

Patients with defects in TAP do not suffer from severe viral infection. This is unexpected but may relate to the fact that they have a normal humoral (antibody) response and significant numbers of T cells, particularly γδT cells, as well as NK cells and neutrophils. Additionally, presentation of some viral antigens is TAP-independent. Another symptom in these patients is necrotizing granulomatous lesions particularly around the nose, resulting in loss of the septum and associated cartilage. Presentation is variable though. The skin ulcers were found to contain macrophages and NK cells.

In terms of biological effects, the proportion of T cells may be reduced in patients with TAP defects, but there is wide variation in phenotype. They tend to have a higher proportion of γδT cells. Interestingly, numbers of NK cells are normal, but they have no cytotoxic activity against the standard class I-negative targets, unless activated by cytokines.

## Proteasome components—LMPs

At the same time that TAPs were discovered, clues to the proteolytic machinery, responsible for breaking the antigenic proteins into peptides, were also provided, by studying mutants over the MHC region. The biochemical prelude to this discovery came in the early 1980s when Monaco and McDevitt described a series of up to16 *low-molecular weight proteins*, LMPs, that mapped to the MHC. Initially, the functions of these proteins were not obvious, but they formed a complex by co-precipitation and varied between different mouse haplotypes, in other words were polymorphic (Monaco and McDevitt 1984). It was subsequently established that genes for two interferon-inducible, catalytic proteasome subunits, LMP2 (PSMB9; β1i) and LMP7 (PSMB8: β5i), were closely linked to the TAP1 and TAP2 genes in both human and mouse MHC (Glynne et al. 1991; Kelly et al. 1991a). As with the TAPs, finding proteasome components encoded in the MHC immediately suggested that the proteasome was responsible for producing the antigenic peptides. Proteasomes are ubiquitous cellular components responsible for continual turnover of proteins in general. However, the MHC-encoded proteasome genes were interferon inducible and, when expressed, replaced constitutive components in a subset of the main structures. Another interferon-inducible subunit PSMB10 (β2i) is encoded outside the MHC on chromosome 16q22.1.

## ERp57 and ERAP1

A number of chaperones are associated with class I as it matures and picks up peptide in the peptide-loading complex. These chaperones serve multiple proteins in the ER and are not dedicated to class I. However, ERp57 is a component of the peptide-loading complex. The protein aids folding of nascent glycoproteins by facilitating disulphide bond isomerisation.

ERAP1 is an amino peptidase. It plays a role in determining the length and sequence of peptides bound and presented by class I allotypes. HLA-B27 is strongly associated with ankylosing spondylitis (Chen et al. 2014), and genetic studies indicated that certain ERAP1 allotypes are more associated with ankylosing spondylitis than others, due to their contribution to peptide trimming (Reeves et al. 2014a, Reeves et al. 2014b). There has been some controversy over this effect as it was difficult to analyse genetically. It was proposed to involve specific *combinations* of different ERAP1 allotypes, making association studies difficult (Reeves et al. 2015; Robinson and Brown 2015).

## TAPASIN

The gene for Tapasin, another component of the antigen processing machinery, is located just outside the MHC region (Ortmann et al. 1997). The product of this gene, which is an additional member of the immunoglobulin gene family, links the peptide transporter to the nascent class I molecule in a complex, the peptide-loading complex (Blees et al. 2017). Optimisation of the MHC class I peptide cargo is proposed to be dependent on Tapasin (Williams et al. 2002). However, class I molecules vary in their dependence on the protein, an effect that has been mapped to specific positions on the HLA molecule (Park et al. 2003) as well as to its conformational flexibility (Garstka et al. 2011). For example, the HLA-B*44 allele, which differs exclusively at position 156, B*44:02 (156Asp), is Tapasin-dependent, whereas other alleles are not (Badrinath et al. 2012). The reason for this is not known but could relate to the fact that some virus products compromise antigen presentation by interacting with the Tapasin protein.

## TAPBPR

There is evidence that the jawed vertebrate genome has undergone duplication at least twice in its evolutionary history, according to the 2R hypothesis. Accordingly, it was demonstrated that there are traces of at least four clusters of genes related to those in the MHC proper on chromosome 6 (Kasahara 1999). One paralogous region, identified by Louis Du Pasquier, was at chromosome position 12p13.3. A gene in this region shared sequence homology with Tapasin, dubbed TAPBPR (Teng et al. 2002). The product of this gene was subsequently shown to be a novel component of the MHC class I presentation pathway, which acts as a peptide exchange catalyst (Hermann et al. 2015; Neerincx and Boyle 2017). TAPBPR interacts with class I in a similar manner to Tapasin but is not complexed to TAP (Jiang et al. 2017). TAPBPR links UDP-glucose:glycoprotein glucosyltransferase 1 onto MHC class I (Neerincx et al. 2017). Tapasin is monomorphic, but TAPBPR is relatively polymorphic and there are a number of common alleles and splice variants in humans (Porter et al. 2014). The relationship between the products of these alleles and MHC Class I loci and alleles is not known. As with Tapasin, it appears that some alleles of class I are dependent, and others independent, of the molecule.

## HLA-DM and DO

The existence of an MHC-encoded factor that was essential for MHC class II antigen presentation was first suggested through the characterisation of mutagenised B lymphoblastoid cells selected for loss of HLA-DR3 reactivity with a DR3-specific antibody, 16:23(Mellins et al. 1990). These cells had normal levels of cell surface DR but were unable to present native antigen to T cells. They possessed class II molecules that were predominantly filled with the CLIP peptide and fell apart in the presence of SDS. The defect was eventually mapped to HLA-DM (Morris et al. 1994), a class II-related molecule previously identified in both mouse and human in screens for expressed genes encoded on cosmids spanning the MHC region (Kelly et al. 1991b; Cho et al. 1991). HLA-DM acts as a peptide editing chaperone stabilising class II whilst exchanging CLIP for antigenic peptide. DM activity is regulated by a second non-classical class II molecule, now called HLA-DO. HLA-DO alpha and beta chains were first identified due to their high homology with human and murine classical class II molecules (Larhammar et al. 1985; Tonnelle et al. 1985; Trowsdale and Kelly 1985). It proved much harder to decipher the role of DO even though the molecule was identified some 6 years before DM. DOA and DOB were not located together in the MHC, and the chains showed different mRNA expression patterns initially suggesting that they would not associate as a pair. DO resides in late endosomal compartments but requires association with DM in the endoplasmic reticulum for correct assembly (Liljedahl et al. 1996). The in vivo role of DO is still debatable, but mechanistically, it functions as a negative regulator of DM activity (van Ham et al. 1997; Denzin et al. 1997). Unlike the transient interactions seen between DM and DR, the DM/DO interaction is very stable, suggesting that DO acts as a competitive inhibitor of DM, a view consistent with the DM/DO crystal structure (Guce et al. 2013).

## Haplotypes and linkage disequilibrium

It has been known for some time that the MHC comprises a large region in linkage disequilibrium (LD), or *polymorphic frozen blocks* (Dawkins et al. 1999). In some populations, for example, combinations of alleles, such as HLA-A1-B8-DR3, are more commonly found together than would be expected based on frequencies of individual alleles in the population. Several mechanisms may be invoked to explain this. First, there may have been insufficient time for recombination after relatively recent expansion of families, in some cases in isolated populations. Another possibility is that recombination ‘cold spots’ in the MHC sequence restrain the level of exchange of alleles between haplotypes. This may be facilitated by a reduced level of pairing at meiosis in a region such as the MHC, where the variation between haplotypes compromises homologous interaction. Indeed, overall recombination is low in the MHC.

An attractive explanation for the high LD is clustering of alleles that encode proteins that work well together and maintenance of functionally coordinated sets of alleles. As discussed above, the discovery of both TAP transporters and LMP proteasome components mapping to the MHC was largely serendipitous and was inspired to some extent by the notion that the MHC was a cluster of genes with inter-related functions. Furthermore, it was proposed that linkage of polymorphic transporters with polymorphic MHC class I genes permitted functional coordination, such that appropriate binding peptides, for the class I molecules linked in *cis*, were pumped by the relevant TAP. This turned out to be the case for rats and chickens, where genes for class I and TAP are in close proximity (Powis et al. 1992; Tregaskes et al. 2016). In the human MHC TAP transporters, genes are separated from the class I genes they serve by the class III region, so this functional integration may not be fully operational in our species. Another consideration is that each individual has two haplotypes, the various components of which must cooperate to some extent.

Recent data add weight to the notion of a high degree of allelic integration on individual haplotypes. It is becoming appreciated that, in addition to protein-coding loci, other polymorphic genomic features are embedded in the MHC complex. One clue to this came from the finding that a single nucleotide polymorphism (SNP) 35 kb upstream of HLA-C associated with levels of mRNA transcript and cell-surface expression (Kulkarni et al. 2011). Binding of microRNA hsa-miR-148 to a variable 3′ untranslated region in HLA-C genes regulated their expression. The conclusion was that expression levels may be regulated both by *cis-* and *trans-*acting factors. A further indication of the integration along haplotypes came from data relating to the way that class I molecules ‘educate’ by interacting with receptors on NK cells. Hydrophobic leader sequences from class I molecules supply peptides that bind HLA-E, which instructs CD94/NKG2A receptors on NK cells. Leader sequences of HLA-B molecules are dimorphic: Those with − 21 methionine (− 21 M) provide peptides that bind HLA-E, whereas − 21 threonine do not. Those haplotypes with -21 M rarely encode the ligands for other NK receptors, namely Bw4+ HLA-B and C2+ HLA-C. From these data, it was proposed that there are two schools of HLA haplotypes; one focused on supplying CD94/NKG2A ligands, and the other, KIR ligands. Individuals with − 21 M had NKG2A+ cells that were more effective than those with only − 21 T (Horowitz et al. 2016). Similarly, HLA-A leader sequences determine expression levels of HLA-E, again influencing interaction with CD94/NKG2A on NK cells and in turn HIV replication (Ramsuran et al. 2018).

## Regulation of transcription and translation

Analysis of mutants has also been productive for identifying regulators of antigen processing and presenting genes. For many years, it was difficult to track down a master regulator of class I. On the other hand, mutants affecting class II were isolated from bare lymphocyte patients (Reith and Mach 2001). The class II master regulator CIITA is a nucleotide-binding domain and leucine-rich repeat receptor (NLR) protein. This large gene was initially found by expression cloning in a class II-deficient cell line (Steimle et al. 1993). Mutants invoking loss of function of CIITA generally result in severe immunodeficiency, a form of bare lymphocyte syndrome. Individuals with BLS have impaired antibody and T cell responses due to the lack of MHC class II expression. Class II transcription requires, in addition to CIITA, at least three additional factors: RFX5, RFX-AP and RFX-ANK. The genes encoding these factors were identified by comparing panels of rare BLS patients and mutant cell lines selected for loss of class II expression. This identified four complementation groups. Transient heterokaryon fusions between these cell lines restored class II expression if the partners harboured defects in different components, thereby defining the four groups. A combination of genetic complementation and protein characterisation eventually identified the genetic lesions, as reviewed in Reith and Mach (2001).

It was only in the last decade that a master transcription regulator for class I was discovered (Kobayashi and Elsen, 2012; Meissner et al. 2010; Neerincx et al. 2013). Like the class II transcription activator, CIITA, NLRC5 is an NLR protein and the two molecules share some homology. In cell lines defective in class I expression, such as mouse melanoma B16F10, over-expressed NLRC5 could restore class I expression. In human cells, over-expression of NLRC5 resulted in induction of non-classical class I genes HLA-E, -F and -G, which normally exhibit a restricted tissue expression. CIITA-dependent activation of HLA class II requires the enhanceosome complex, which binds to a motif, SXY, in the promoter of the gene. NLRCF probably interacts with a similar module upstream of class I genes.

Confirmation of the role of NLRC5 in regulation of MHC class I was provided by studying *Nlrc5*-deficient knock-out mice (Staehli et al. 2012). The data showed that MHC class I was downregulated, exhibiting the greatest effect in cells in the immune system. However, treatment of these mice with inflammatory stimuli, such as interferon or LPS, resulted in significant class I expression, indicating that *Nlrc5*-independent mechanisms must exist to regulate MHC class I.

Recent work suggests that there is still more to learn about variation in HLA expression. For example, the Anderson laboratory identified an elaborate system regulating forms of HLA-C, which is specific to natural killer cells, and could relate to NK cell licencing or education (Li et al. 2018). Other work suggests that, in addition to allelic differences, levels of class I should be taken into account in relation to autoimmunity, infection and transplantation (Apps et al. 2013; Kaur et al. 2017; Li et al. 2018; Petersdorf et al. 2014).

## Screening for novel components

New screening techniques have the potential to identify novel factors involved in MHC biology. For example, forward genetic screens involving retroviral insertional mutagenesis or random CRISPR/Cas9 targeting of a haploid human cell line KBM7 implicate TXNDC11 as a novel factor required for MHC class I endoplasmic reticulum-associated protein degradation (ERAD) (Timms et al. 2016). In addition, a genome-wide RNAi screen was used to identify pathways regulating MHC class II antigen presentation (Paul et al. 2011).

## Conclusion

Immunogenetics has played a predominant role in uncovering many of the fundamentals of antigen processing and presentation. In this review, we have sketched out some of the history and background to the genetics, focussing mainly on the human system. Other contributions to this volume of Immunogenetics may be consulted for more in-depth coverage of individual components of antigen processing and presentation.

## Footnotes

1
https://www.ebi.ac.uk/cgi-bin/ipd/imgt/hla/fetch_cell.cgi?10972
